# Exploring the Association between Attachment Style, Psychological Well-Being, and Relationship Status in Young Adults and Adults—A Cross-Sectional Study

**DOI:** 10.3390/ejihpe13030040

**Published:** 2023-02-24

**Authors:** Elisabetta Sagone, Elena Commodari, Maria Luisa Indiana, Valentina Lucia La Rosa

**Affiliations:** Department of Educational Sciences, University of Catania, Via Biblioteca 4, 95124 Catania, Italy

**Keywords:** attachment, well-being, close relationships, singleness

## Abstract

*Background:* This study aimed to analyze the associations of adult attachment styles with psychological well-being in relation to age groups (young adults vs adults) and relationship status (singleness vs close relationships). *Method*: The study sample consisted of 393 Italian young adults and adults, aged 18 to 62 years, with stable close relationships (*n* = 219) or identified in this study as singles (*n* = 174). The Psychological Well-being Scale was used to analyze psychological well-being, and the Attachment Style Questionnaire was chosen to evaluate adult attachment dimensions. *Results:* Individuals with stable close relationships reported higher levels of psychological well-being than singles. Furthermore, compared to people with stable close relationships, singles had an attachment style associated with discomfort with closeness, relationships as secondary, and avoidance. Finally, in single people, psychological well-being was moderately and positively predicted by attachment style characterized by confidence but strongly and negatively by attachment characterized by the need for approval. Regarding individuals with stable relationships, psychological well-being was strongly and negatively predicted by attachment style characterized by the need for approval. Conclusions: In adult attachment styles, close relationships can be viewed as a protective factor for long-term emotional stability and psychological well-being.

## 1. Introduction

### 1.1. Attachment Theory and Romantic Relationships

Notably, affective experiences in childhood have a relevant impact on the type and quality of relationships that individuals develop as adults. Therefore, a corpus of attachment theories provides significant frameworks for examining the quality of close relationships across one’s lifespan [1]. Generally, according to a categorical approach, widely developed starting from Bowlby’s model, attachment based on security is defined as the confidence in the emotional availability and accessibility of primary figures perceived as a secure base for restoring emotional balance during distressed and needed situations [2]; attachment characterized by anxiety is defined as the perceived inability to face challenges on one’s own, which increases the desire for interpersonal closeness, love, and growth support, despite the inconsistent behavior of the attachment figures [3]; lastly, attachment linked to avoidance is characterized by the difficulty with interpersonal relationship and worry of trusting people, and a significant emphasis on autonomy and independence, useful to prevent the emotions evoked by rejection by the others [4]. According to Bowlby’s developmental attachment theory [5], early caregiving experiences become generally stable internal working attachment models over time. They frequently guide people as they look to form relationships, particularly intimate ones, later in life [6,7]. People can develop a stable attachment with a sufficient amount of self-esteem, emotional stability, and a favorable perception of both themselves and others if attachment-related mental representations are positive. These positively impact people’s cognition, emotion control, and behavior, which in turn positively impact their well-being [7,8].

In contrast, people might adopt attachments defined by two types of insecure strategies if attachment experiences with primary figures are insufficient to create secure internal working models [7]. Typically present in people with an anxious attachment [7,9,10], hyperactivation strategies include a high need for care, persistent search for proximity and protection, rumination, and an intense worry about abandonment. On the contrary, frequently present in people with avoidant attachment, deactivation strategies are characterized by high self-reliance, aloofness, emotional distance, alienation from others, and inhibition of the desire for attachment and attachment-related thoughts [8,9,10]. The debate has questioned this type of categorical analysis of attachment about whether differences among individuals are best captured using categorical or continuous models, and more recent studies indicate that individual differences in styles of attachment appear more consistent with the multidimensional and continuous models rather than with the categorical ones [11]. To cite one of the most relevant contributions to this debate [12,13,14], Fraley and colleagues [11] revisited the taxonomy of adult attachment not only at the level of global attachment representations but also in the context of specific relationships (e.g., attachment with parents and romantic partner) and demonstrated that dimensional models of attachment style that take into consideration continuous dimensions rather than categorical ones may be better adequate for conceptualizing and measuring the differences among individuals across multiple levels of analysis of attachment in relationship-specific domains.

The models of adult attachment used in this study as a framework for conceptualizing the differences in romantic attachment are represented by Hazan and Shaver’s adult attachment prototypes [14] chosen for the content of the attachment styles with a partner, and by Feeney et al.’s model of attachment [6] selected for the dimensional measurement. According to Hazan and Shaver, romantic love is an attachment process experienced differently by people due to variations in their attachment histories. They described why both functional and dysfunctional expressions of love develop as justifiable adjustments to particular social circumstances [15]. In line with the previous attachment classification suggested by Bowlby and successively by Ainsworth et al. [16], Hazan and Shaver analyzed the relationships between the perception of love and attachment. They wrote that “secure types should believe in enduring love, generally find others trustworthy, and have confidence that the self is likeable. Avoidant types should be more doubtful of romantic love’s existence or durability and believe they do not need a love partner to be happy. Anxious/ambivalent types should fall in love frequently and easily but have difficulty finding true love. They should also have more doubts than the other two types because, unlike avoidant respondents, they do not repress or attempt to hide feelings of insecurity” [15]. To understand the relationship-specific attachment and analyze its dimensions according to Feeney’s model, we used the Attachment Style Questionnaire (ASQ) because it developed a more precise measure of attachment that extended beyond category-based measures and attempted to resolve the limitations observed in categorical measures. The five dimensions are called “discomfort with closeness” (referring to difficulty trusting others), “relationships as secondary” (related to the belief that achievement is more important than relationships with others), “need for approval” (focused on validation from others and fear of rejection and avoiding doing things that other people will not like), “preoccupation with relationships” (referring to the worry of being abandoned and not making it on your own), and confidence about themselves and others. People with high confidence find it easy to trust others and to get along with others, and they don’t mind depending on others or having other people who rely on them; this last dimension is strictly related to secure-romantic attachment, while the other four dimensions are linked to the romantic-insecure attachment.

### 1.2. Romantic Attachment, Relationship Status, and Psychological Well-Being

According to the literature on the topic, adult attachment orientation may vary over one’s lifespan [17]. Several authors have suggested that attachment styles differ with age, particularly between young and old individuals [18,19,20]. Specifically, adolescents and young adults would experience more attachment anxiety, while older and middle-aged adults would experience less (after the development of enduring intimate relationships) [17,18,21]. Furthermore, a higher level of attachment avoidance may accompany changes in young adulthood [17].

In general, it is well established that attachment style differs with age, particularly in early adulthood, such that anxiety tends to be higher in younger adults compared to older adults and avoidance tends to be higher in middle-aged adults compared to younger adults [17,18]. However, several variables can be associated with adult attachment styles in one’s lifespan. In particular, people in romantic relationships feel more secure than those who are single [22]. In fact, individuals with a secure attachment may be more likely to have romantic relationships because they exhibit many of the characteristics that make a long-term partner desirable (e.g., attentiveness, warmth, and sensitivity) [23]. From this perspective, attachment style appears to be significantly correlated with relationship status. For example, Brauer et al. [24,25] showed that attachment predicts current relationship status and prior relationship status over one’s lifetime.

Several studies also demonstrated a positive relationship between secure attachment and psychological adjustment of individuals, positive emotions, greater search for social support, and higher satisfaction in romantic relationships [26,27,28,29]. In contrast, individuals characterized by an anxious attachment perceived more conflict in their relationships [30]. Recently, according to Nourialeagha et al. [31], anxiety-avoidance attachment is negatively related to a sense of gratitude and lower levels of psychological well-being; secure attachment is positively associated with both outcomes. Additionally, individuals who exhibited avoidant attachment had a positive model of themselves but a negative model of others; the first allows individuals to feel confident facing the obstacles of their environment, while the second is linked to doubt, low levels of sociability, and lower warmth in interpersonal relationships [7,32]. Concerning the differences in personality traits, people with anxious and avoidant attachment displayed high neuroticism, low extraversion, and a lower level of friendliness than those with secure attachment [33,34]. During childhood, Fransson et al. [35] found a direct association between secure attachment, extraversion, and openness to experience. Furthermore, Both and Best [36] observed that people with secure attachment were characterized by low neuroticism (depression, anxiety, or self-consciousness) and high activity. In contrast, low depression and low agreeability were the typical characteristics of people with anxious attachment.

Furthermore, the literature widely supported the positive connection between secure attachment and individual well-being, and researchers have often focused their investigations on the subjective component of well-being (e.g., satisfaction with life, positive and negative affect) [37,38] rather than on the psychological components [39,40,41,42]. Specifically, Karreman and Vingerhoets [41] found that preoccupied attachment was linked to lower levels of well-being, whereas secure and dismissing attachment were related to higher levels of well-being. According to Diehl et al. [40], people with a secure attachment positively perceived their family of origin and their current family and showed high levels of personality traits such as dominance, sociality, social presence, self-acceptance, and empathy (measured by the California Personality Inventory-CPI [43]): so, these individuals reported a greater level of self-confidence, psychological well-being, and functioning in the social world than individuals with insecure attachment. In a recent study by Marrero-Quevedo et al. [42], correlations between secure attachment and dimensions of psychological well-being were positive. In contrast, correlations between avoidant/anxious attachment and psychological well-being were negative.

Regarding the bond between psychological well-being and type of attachment, some studies have discovered that the attachment styles of individuals play a significant role in influencing well-being [20,31,41,44]. According to these findings, insecure attachment styles (such as anxious and avoidant attachment) are inversely associated with overall well-being, whereas attachment security is positively correlated [7,41,42,44,45]. Specifically, people who had secure attachment experiences displayed good interpersonal relationships, high degrees of autonomy, adequate environmental mastery, a sense of purpose in life, and high levels of self-acceptance [46]; in addition, high levels of autonomy, personal growth (in terms of competence), and positive relationships with others are related to the secure attachment of an individual [47,48]. On the contrary, those who expressed avoidant attachment had trouble managing their interactions with others and their surroundings, and they also indicated less self-acceptance than the other groups. The mediational roles of dispositional mindfulness, psychological inflexibility, and resilience in the relationship between attachment styles and psychological well-being were examined in the Italian context by Calvo et al. [45]. The data suggested that lower levels of psychological well-being were correlated with higher levels of attachment anxiety and avoidance. Attachment anxiety and avoidance can severely decrease people’s well-being by raising psychological rigidity, lowering resilience, and lowering expressed awareness. At the same time, individuals with anxious attachment showed low levels of autonomy and self-acceptance [42,49]. Specifically, Kawamoto [50] underlined the effect of attachment on the development of self-concept and self-esteem in a large sample of Japanese adolescents and young adults, indicating that individuals characterized by anxious attachment reported low levels of self-esteem.

Psychological well-being is also significantly influenced by the quality of the individual’s relationships. In particular, stable and positive romantic relationships, which in turn are promoted by a secure attachment style, are associated with higher levels of psychological well-being and lower levels of distress and psychological discomfort [49,51].

These findings highlighted the need to deepen the association of attachment styles with psychological well-being based on differences related to age and relationship status. Therefore, the rationale of this research is to analyze the associations of adult attachment styles with psychological well-being in young adults and adults, and in single people and those with a stable close relationship.

Specifically, we hypothesized that:

**H_1_:**  *Individuals with stable close relationships score higher in psychological well-being than singles*;

**H_2_:**  *Individuals with stable close relationships report an attachment linked to confidence more than singles*;

**H_3_:**  *Individuals with an attachment style characterized by confidence have a higher likelihood of having a stable relationship than those characterized by discomfort with closeness, need for approval, preoccupation with relationships, and relationships as secondary*;

**H_4_:**  *Adults with stable relationships score higher in psychological well-being than single young adults*;

**H_5_:**  *Adults with stable relationships report an attachment linked to confidence more than single young adults*;

**H_6_**  *Primary aim:* *The more individuals display an attachment style linked to confidence, the higher they score in psychological well-being, compared to people with attachment styles characterized by discomfort with closeness, need for approval, preoccupation with relationships, and relationships as secondary* **.**

## 2. Materials and Methods

### 2.1. Sample

The sample consisted of 237 young adults (157 female students and 80 male students) and 156 adults (68 men and 88 women) recruited to participate in this study voluntarily, including individuals with stable close relationships (*n* = 219) and single individuals (*n* = 174). The participants ranged from 18 to 62 years, and two age groups were created: young adults (18–30 years old; *M* = 22.4, *SD* = 2.8) and adults (32–62 years old; *M* = 43.5, *SD* = 3.7).

The young adults were randomly enrolled in bachelor’s and master’s degree courses in Psychology and Pedagogy at the Department of Educational Sciences of the University of Catania (Italy). Specifically, researchers reached out to students in different areas of the Department, such as the library, cafeteria, and classrooms. First, they were informed about the study’s objectives and then asked to complete the questionnaires. Respondents who agreed to participate were given a questionnaire to complete and return.

In the same period, adults were randomly chosen from three association centers (sports centers, entertainment centers, and bookshops, respectively) and invited to complete the same questionnaire as the university students.

The selection criteria for including participants in this study were those who had experienced stable and lasting close relationships (for more than three years that are still present) or were single. The distribution of the sample for gender and age groups within the variable of “close relationships” was as follows: 79 single men and 69 men with close relationships, 95 single women and 150 women with intimate relationships (*Χ*^2^ = 7.974, *p* = 0.005); 118 single young adults and 119 young adults with close relationships, 56 single adults and 100 adults with close relationships (*Χ*^2^ = 7.358, *p* = 0.007).

### 2.2. Measures and Procedures

The present study was a component of larger research looking at things that affect young adults’ and adults’ psychological health. It was carried out according to the Declaration of Helsinki guidelines and was approved by the Board of Psychology Research of the Department of Educational Sciences of the University of Catania (date of approval: 13 January 2021). Furthermore, the researchers respected the Ethics Code for Italian psychologists (L. 18.02.1989, n.56), the Legislative Decree for the privacy of provided data (DLGS 196/2003), and the Ethics Code for Psychological Research (27 March 2015) established by the Italian Psychologists Association.

An anonymous questionnaire was used to collect data from university students (young adults) attending bachelor’s and master’s degree courses in Psychology and Pedagogy before the COVID-19 outbreak. In addition, the same questionnaire was administered to adults during recreational activities in some associations in the Sicilian social context. At the end of the structured questionnaire, participants were asked to point out the following independent variables: gender, age, and close relationships. The investigation was started after receiving informed consent. The questionnaire comprised the following validated scales: the Psychological Well-being Scales [52] and the Attachment Style Questionnaire [6].

#### 2.2.1. Psychological Well-Being Scales (PWB)

The Italian short form of the PWB [53] was used to measure the main dimensions in the eudaimonic perspective of psychological well-being (autonomy, environmental mastery, positive relationships, personal growth, purpose in life, and self-acceptance). These 18-item self-report questionnaires required respondents to rate their level of agreement or disagreement with each statement on a six-point Likert scale, with 1 indicating “strong disagreement” and 6 indicating “strong agreement”. Six subscales were created from the 18 items: (1) autonomy: e.g., *I tend to be influenced by people who have a strong personality*; (2) environmental mastery: e.g., *I am very good at managing the responsibilities of daily life*; (3) purpose in life: e.g., *I’m one of those people with so many projects in life*; (4) positive relations with others: e.g., *Maintaining stable friendships over time has been difficult and frustrating for me*; (5) personal growth: e.g., *For me, life has been a continuous process of learning, change and growth*; (6) self-acceptance: e.g., *I don’t feel satisfied with the results in my life*. The responses were computed for each of the six subscales (approximately 50% of the responses were reverse-scored), and high scores for each scale indicated high levels of psychological well-being. By summing each item from the six dimensions, a total PWB score was determined [54,55,56]. The PWB score in the current study displayed strong internal consistency (α = 0.82). Furthermore, the one-factor model of the Italian PWB has an adequate to good statistical fit, according to a prior validation study [53].

#### 2.2.2. Attachment Style Questionnaire (ASQ)

The ASQ [6] was used to explore adult attachment using continuous measures (confidence, discomfort with closeness, need for approval, preoccupation with relationships, and relationships as secondary). A six-point Likert scale, ranging from 1 (completely disagree) to 6 (totally agree), was used by participants to score the degree to which each item accurately characterized their emotions and actions in close relationships. The five-factor model was validated for the Italian version of the ASQ [57]: (1) *confidence* (e.g., I am confident that other people will like and respect me); (2) *discomfort with closeness* (e.g., I prefer to depend on myself rather than other people; (3) *need for approval and confirmation by others* (e.g., It’s important to me that others like me); (4) *preoccupation with relationships* (e.g., I find that others are reluctant to get as close as I would like; (5) *relationships as secondary* (e.g., Doing your best is more important than getting on with others). All subscales showed good internal consistency in our sample (confidence: α = 0.79; discomfort with closeness: α = 0.69; need for approval: α = 0.72; preoccupation with relationships: α = 0.78; relationships as secondary: α = 0.86).

### 2.3. Data Analysis

The sample size was calculated using G*Power 3.1 [58]. Taking into account a power of 0.90, 7 predictors (i.e., the maximum number of predictors included in multiple linear regression), and α = 0.05, a sample of 73 subjects for each group is adequate to detect a minimum effect size of f^2^ = 0.15, which is considered a medium effect [59].

All the analyses were performed using The Statistical Package for the Social Sciences (SPSS) version 25.0 (IBM Corporation, Armonk, NY, USA). Mean (M) ± standard deviation (SD) was used for continuous variables, while categorical variables were expressed as frequencies and percentages. Independent-samples *t*-tests and one-way ANOVA were run to test study hypotheses. The magnitude of the differences between the means was assessed by calculating the effect size through Hedge’s formula for *t*-tests [60] and eta squared (η^2^) for ANOVA. Direct logistic regression was performed to assess the impact of attachment dimensions on the likelihood that respondents would have or not have a close relationship. Finally, multiple regression models were run to investigate the impact of adult attachment style on psychological well-being in single people and people in close relationships. A *p*-value < 0.05 was considered significant.

## 3. Results

Regarding hypothesis **H_1_**, differences were observed for the type of relationships in the overall PWB score (*t* = −2.179, *p* = 0.030); individuals with stable close relationships scored higher (*M* = 79.41, *SD* = 7.9) in psychological well-being than singles (*M* = 77.40, *SD* = 10.3). However, the effect size was small (*g* = 0.221).

Concerning hypothesis **H_2_**, significant differences were observed in adult attachment for the type of relationships: singles scored higher than those with stable and close relationships with discomfort with closeness (*t* = 3.535, *p* < 0.001, *g* = 0.358) and secondary relationships (*t* = 2.180, *p* = 0.030, *g* = 0.221) (Table 1). However, the effect sizes were small.

Regarding the impact of attachment dimensions on the likelihood of having or not having a close relationship, the logistic regression model partially confirmed hypothesis **H_3_**. As shown in Table 2, an attachment style characterized by discomfort with closeness reduces the odds of having a close relationship by 0.953. In more detail, the odds of having an intimate relationship are 0.949 times lower for individuals with an attachment style characterized by discomfort with closeness (OR = 0.953, 95% CI = [0.919, 0.989], *p* = 0.012). Furthermore, the odds of having a close relationship are 1.677 times higher for females than males (OR = 1.677, 95% CI = [1.084, 2.593], *p* = 0.020).

Cross-referencing the two independent variables (age groups and close relationships), results showed that:

**H_4_:**  *Adults with stable relationships scored higher (M = 79.74, SD = 8.01) in psychological well-being than single young adults (M = 76.86, SD = 10.1) (F = 2.790, p = 0.030). The effect size was small (η^2^ = 0.016)*;

**H_5_:**  *The single young adults reported higher scores of discomfort with closeness (M = 37.12, SD = 6.80) than the adults with close relationships (M = 32.68, SD = 5.03, F = 9.40, p < 0.001). The effect size was medium (η^2^ = 0.068). The single young adults scored higher in need for approval (F = 5.05, p = 0.002) and preoccupation with relationships (F = 5.71, p = 0.001) than the single adults. The effect sizes were small. Finally, single adults reported higher scores of confidence (M = 31.86, SD = 4.80) than single young adults (M = 29.57, SD = 5.4; F = 3.58, p = 0.014) with a small effect size (η^2^ = 0.027)*.

Concerning **H_6_**, as reported in Table 3, PWB (total score) was positively correlated with confidence (*r* = 0.45, *p* < 0.001) and negatively with discomfort with closeness (*r* = −0.38, *p* < 0.001), relationships as secondary (*r* = −0.32, *p* < 0.001), need for approval (*r* = −0.68, *p* < 0.001), and preoccupation with relationships (*r* = −0.41, *p* < 0.001) in the group of singles. Similarly, PWB (total score) was positively correlated with confidence (*r* = 0.25, *p* < 0.001) and negatively with discomfort with closeness (*r* = −0.37, *p* < 0.001), relationships as secondary (*r* = −0.31, *p* < 0.001), need for approval (*r* = −0.54, *p* < 0.001), and preoccupation with relationships (*r* = −0.32, *p* < 0.001) in the group of close relationships.

As shown in Table 4, the analysis of multiple regressions partially confirms the validity of the hypothesized model for the group of singles, according to which psychological well-being was positively associated with attachment styles characterized by confidence and negatively with attachment styles characterized by the need for approval.

As shown in Table 5, the analysis of multiple regressions partially confirms the validity of the hypothesized model for the group of participants with stable and close relationships, according to which psychological well-being is negatively associated with attachment styles characterized by need for approval, discomfort with closeness, and relationships considered as secondary.

## 4. Discussion

This study analyzed the association between adult attachment styles and psychological well-being. Specifically, we investigated these variables in both singles and people with stable and close relationships, as well as in young and adult people.

First, our results showed the association between romantic relationships and an individual’s psychological well-being, although the effect size was not large in our sample. Individuals with stable close relationships in our sample reported higher scores in psychological well-being than singles. In this regard, the data from the literature have clearly shown the association between stable romantic relationships and mental health in young adults and adults. Attachment theory considers the “capacity to make intimate emotional bonds with other individuals… as a principal feature of effective personality functioning and mental health” [2]. Confirming this, several studies underlined that successful romantic relationships could support well-being and happiness among university students.

Furthermore, stable and positive romantic relationships are associated with lower levels of psychopathology, a good view of oneself, effective emotional regulation, and higher self-esteem [7]. This finding can also be explained by referring to the literature pointing out that relationships are essential in regulating stress [61]. More specifically, it seems that the quality of affective relationships exerts an essential impact on the physiological systems of emotion regulation (e.g., the endocrine system, the autonomic nervous system, and the immune system), allowing a better stress response and, thus, greater psychological well-being.

Interestingly, according to our findings, individuals with an attachment style characterized by discomfort with closeness are more likely to be single and not establish stable romantic relationships. At the same time, singles reported higher scores of discomfort with closeness and relationships as secondary than participants with stable and close relationships. The data in the literature on attachment characteristics in single people compared to those in a relationship are quite conflicting. In a long-term study including 144 dating couples, Simpson [62] investigated the effects of secure, anxious, and avoidant attachment styles on romantic relationships. Compared to the anxious or avoidant attachment styles, the secure attachment style was related to higher levels of relationship interdependence, commitment, trust, and satisfaction in both men and women. Less frequent positive emotions and more frequent negative emotions in the relationship were linked to anxious and avoidant styles, whereas the opposite was true of the secure attachment. In 6-month follow-up interviews, it was discovered that avoidant men considerably suffered less post-dissolution emotional suffering than others.

Regarding singles, according to some authors, single people tend to be more avoidant than people in relationships, favoring more independence and self-reliance [63]. Another hypothesis is that singles report higher levels of anxiety attachment with frequent rejection by partners precisely because of their anxiety and intrusiveness. Finally, according to other authors such as De Paulo [64], single people are as likely as people in a stable relationship to exhibit a secure attachment. Still, their attachment figures are people other than a romantic partner (friends, siblings, etc.). The results of this study seem to be in line with the first hypothesis of the literature, namely that singles report to a greater extent than people in stable relationships an avoidant attachment characterized mainly by discomfort with closeness and relationships seen as secondary. In addition, according to Sousa-Gomes et al. [65], less ability of self-regulation predicts less security of bonds and high insecurity in terms of dependence, ambivalence, and avoidance. However, additional research is required to better understand the connection between these variables.

In line with our results, a recent study by Calvo et al. [55] in a sample of Italian adults showed a link between lower levels of psychological well-being and avoidant and anxious attachment [7,8]. In this regard, several studies confirmed that attachment patterns are closely associated with psychological well-being. In general, the psychological well-being of individuals with secure attachment is often higher than those with insecure attachment [42]. On the other hand, those with insecure attachment usually report worse well-being, lower self-esteem, and more significant psychological distress [50]. As mentioned earlier, attachment style can serve as a means of emotion regulation, thus contributing to psychological well-being. In this sense, anxious and avoidant individuals have been reported to have higher cortisol levels in the context of relational stress [66]. Individuals with high insecurity in attachment and low intimacy perceived low satisfaction levels in their relationships with partners and increased depressive symptoms [67]. Furthermore, avoidant individuals showed higher autonomic nervous system activity and poor immune function [68].

According to our hypotheses, in singles, psychological well-being was positively associated with attachment styles characterized by confidence and negatively with attachment styles characterized by the need for approval. Regarding individuals with stable relationships, psychological well-being was positively associated with attachment styles characterized by confidence and negatively with attachment styles characterized by need for approval, discomfort with closeness, and relationships considered as secondary. Moreover, age was significantly associated with psychological well-being in both groups. In particular, older individuals reported a lower psychological well-being than younger participants, as in other similar studies [42]. Finally, gender was found to be significantly associated with psychological well-being in individuals in stable relationships. Specifically, females tend to report lower psychological well-being scores. However, this finding should be considered cautiously due to the clear prevalence of women in the study sample.

Need for approval appears to be the dimension of attachment most significantly related to psychological well-being in our sample, as we found a negative association in both singles and people in stable relationships. This finding perfectly aligns with the literature on the topic, which shows that individuals with high adult attachment anxiety show an excessive need for approval with a significant negative impact on general psychological well-being [69].

### Study Limitations

The current study has some limitations to take into account. First, we could not add other participants to the sample due to the lockdown by COVID-19. However, the sample size calculation showed our sample’s suitability to detect a medium effect. Second, the sample was unbalanced with respect to gender with a clear predominance of females over males. Furthermore, the study’s cross-sectional nature does not allow us to test the exact causal relationship between variables; longitudinal studies with large samples would be needed for this purpose. Another limitation is the use of quantitative data and non-qualitative responses regarding the perception or level of satisfaction with the close relationships of the participants.

Furthermore, we collected only information on the presence or absence of close relationships for more than three years that are currently still present. Probably, the length and stability of relationships among the young adults today are considered highly variable conditions if compared to those of the adults, and this explanation is proper in relation to lifestyles in Italy [70,71]. Another limitation of this study is that it did not investigate specific variables such as reasons behind being single or the duration of relationship status. Differences between heterosexual and homosexual or polyamorous couples could also not be assessed because of the few cases detected. Therefore, future studies will need to consider these factors to close the gap in the results of this study.

## 5. Conclusions

In conclusion, the primary goal of this study was to explore the association between attachment styles and psychological well-being in young adults and adults with or without stable relationships. These findings underline the primary role of attachment styles and relational patterns in affecting an individual’s psychological well-being, as widely reported in the literature. At the same time, they address future research to clarify better these dimensions’ role in psychological well-being and emotional strength during one’s lifespan. Therefore, this study fits into the field of research on attachment from a eudaimonic perspective that focuses on the individual’s self-realization and factors that contribute to psychological well-being. From this perspective, attachment styles and relationships are analyzed from the perspective of well-being and not psychopathology, unlike the traditional literature on the topic.

In conclusion, the findings of this study encourage further studies with large samples drawn from various socio-cultural situations in order to analyze the perceived quality of the close relationships in terms of high versus low levels of satisfaction with a partner, and to estimate the influence of other variables (e.g., quality of intimate relationships together with personality traits and search of support) in psychological well-being.

## Figures and Tables

**Table 1:** Differences for the type of relationships according to ASQ scores.

Variable	Type of Relationship	*N*	*M*	*SD*	*t*	*p*	Hedges’ *g*
Confidence	I	174	30.30	5.32			
	II	219	30.72	4.58	−0.81	0.418	−0.084
Discomfort with Closeness	I	174	35.95	7.06			
	II	219	33.58	6.23	3.535	< 0.001	0.358
Relationships as Secondary	I	174	15.41	4.90			
	II	219	14.34	4.78	2.180	0.030	0.221
Need for approval	I	174	19.85	6.62			
	II	219	19.10	6.43	1.140	0.255	0.116
Preoccupation with relationships	I	174	28.39	6.94			
	II	219	27.80	7.06	0.823	0.411	0.083

*Note*. I = group of singles; II = group of stable and close relationships.

**Table 2:** Logistic regression predicting the likelihood of having a close relationship.

	B	SE	Wald	df	p	Odds Ratio	95% CI for Odds Ratio	
							Lower	Upper
Gender	0.517	0.222	5.398	1	0.020	1.677	1.084	2.593
Age	0.012	0.008	2.148	1	0.143	1.013	0.996	1.030
Confidence	−0.011	0.023	0.213	1	0.644	0.989	0.946	1.035
Discomfort with Closeness	−0.048	0.019	6.368	1	0.012	0.953	0.919	0.989
Relationships as Secondary	−0.020	0.024	0.692	1	0.406	0.980	0.935	1.028
Need for approval	0.004	0.022	0.041	1	0.839	1.004	0.963	1.048
Preoccupation with relationships	0.003	0.019	0.034	1	0.854	1.003	0.967	1.041
Constant	1.614	1.166	1.918	1	0.166	5.024		

**Table 3:** Correlations table for the two groups (single vs close relationship).

Variable	1	2	3	4	5	6	7
1. Age	-	0.11	−0.15 *	−0.04	−0.22 **	−0.15 *	−0.13 *
2. Confidence	0.25 ***	-	−0.33 ***	−0.12	−0.24 ***	−0.26 ***	0.25 ***
3. Discomfort with Closeness	−0.22 **	−0.30 ***	-	0.29 ***	0.44 ***	0.40 ***	−0.37 ***
4. Relationships as Secondary	0.02	−0.31 ***	0.34 ***	-	0.33 ***	0.13	−0.31 ***
5. Need for approval	−0.20 **	−0.32 ***	0.37 ***	0.29 ***	-	0.55 ***	−0.54 ***
6. Preoccupation with relationships	−0.21 **	−0.02	0.35 ***	0.09	0.61 ***	-	−0.32 ***
7. PWB total	0.06	0.45 ***	−0.38 ***	−0.32 ***	−0.68 ***	−0.41 ***	-

*Note*. Correlations for single people (n = 174) are reported below the diagonal. Correlations for people with close relationships (n = 219) are reported above the diagonal. PWB = Psychological Well-Being Scales. * *p* < 0.05. ** *p* < 0.01. *** *p* < 0.001.

**Table 4:** Multiple regressions for psychological well-being (PWB)—singles group.

Variable	*B*	*SE*	*β*	*t*	*R* ^2^	Δ*R* ^2^
					0.55	0.54 ***
Constant	90.57 ***	5.65		16.03		
Gender	0.18	1.14	0.009	0.16		
Age	−0.12 **	0.04	−0.15 **	−2.74		
Confidence	0.53 ***	0.12	0.27 ***	4.52		
Discomfort with Closeness	−0.13	0.09	−0.09	−1.52		
Relationships as Secondary	−0.08	0.12	−0.04	−0.67		
Need for approval	−0.83 ***	0.12	−0.54 ***	−7.16		
Preoccupation with relationships	−0.10	0.10	−0.07	−0.98		

** *p* < 0.01. *** *p* < 0.001.

**Table 5:** Multiple regressions for psychological well-being (PWB)—group of close relationships.

Variable	*B*	*SE*	*β*	*t*	*R* ^2^	Δ*R* ^2^
					0.43	0.41 ***
Constant	100.92 ***	4.83		20.88		
Gender	−2.69 **	0.95	−0.16 **	−2.83		
Age	−0.17 ***	0.03	−0.27 ***	−5.14		
Confidence	0.20 *	0.09	0.12 *	2.09		
Discomfort with Closeness	−0.20 *	0.08	−0.15 *	−2.48		
Relationships as Secondary	−0.24 *	0.09	−0.15 *	−2.53		
Need for approval	−0.59 ***	0.08	−0.48 ***	−7.02		
Preoccupation with relationships	−0.04	0.07	−0.03	−0.50		

* *p* < 0.05. ** *p* < 0.01. *** *p* < 0.001.

## Data Availability

The data presented in this study are available on request from the corresponding author. The data are not publicly available due to Department policy.
